# P-1140. Antimicrobial Stewardship Curriculum Development for Pediatric Residents

**DOI:** 10.1093/ofid/ofae631.1327

**Published:** 2025-01-29

**Authors:** Matthew Sattler, Monica Abdelnour, Christine Lockowitz, Evan E Facer, Sara Greer, Katie Wolfe, Jason G Newland

**Affiliations:** Washington University in St. Louis School of Medicine, Department of Pediatrics, Division of Infectious Diseases, Saint Louis, Missouri; Washington University, St. Louis, Missouri; St. Louis Children's Hospital, St. Louis, Missouri; St. Louis Children's Hospital, St. Louis, Missouri; Washington University in St. Louis School of Medicine, St. Louis, Missouri; Washington University in St. Louis School of Medicine, St. Louis, Missouri; Washington University in St. Louis School of Medicine, St. Louis, Missouri

## Abstract

**Background:**

Although both the Centers for Disease Control and Prevention and the World Health Organization identify education as a core component of antimicrobial stewardship (AS) programs, there is little data to inform AS curriculum development for pediatric residents.

Demographics of Resident Survey Respondents.

**Figure:**
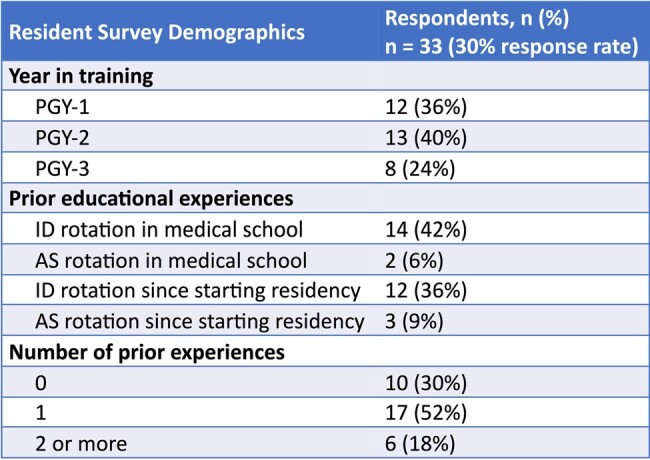

Resident respondents are categorized by their year in training, their prior educational experiences, and their number of prior educational experiences.

PGY: post-graduate year, ID: infectious diseases, AS: antimicrobial stewardship

**Methods:**

Pediatric residents at a single academic tertiary-care children’s hospital were surveyed to identify prior AS educational experiences and usefulness of additional education on different AS domains, among other topics. Content experts (pediatric infectious diseases [ID] faculty, fellows, advanced practice providers, and pharmacists) at the same institution were surveyed to determine what material should be included in an AS curriculum for pediatric residents. A topic was considered to have achieved consensus if ≥80% of respondents identified having knowledge of this topic as “very” or “extremely” important for graduating pediatric residents.

**Figure:**
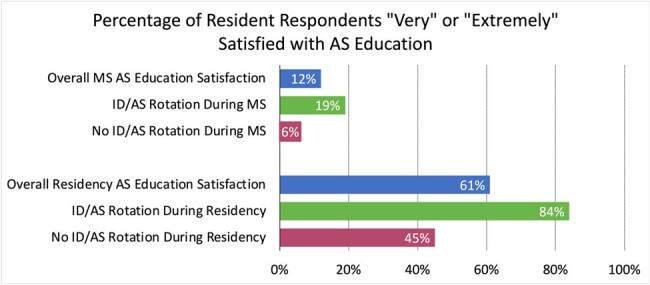

The difference in satisfaction in AS education since starting residency training between those who had completed an ID/AS rotation and those who had not was statistically significant (OR 6.1, 95% CI 1.2-52.6, p=0.03). There was no statistically significant difference in MS AS education when comparing those who completed a rotation in medical school to those who did not (OR 3.3, 95% CI 0.34-102.8, p=0.3).

MS: medical school, AS: antimicrobial stewardship, ID: infectious diseases

**Results:**

33/110 (30%) of pediatric residents responded to the resident survey. The domains on which most residents identified that additional education would be “very” or “extremely” useful were spectrum of activity (97%), duration of therapy (94%), and empiric therapy (94%). Respondents who had completed an ID or AS rotation as a medical student or resident were more likely to identify additional education on antimicrobial resistance (AMR) as “very” or “extremely” useful than those who had never completed an ID or AS rotation (87% versus 40%, OR 8.90, 95% CI 1.61-63.0, p=0.01).

26/26 (100%) of content experts responded to the content expert survey. Overall, 39 of 105 topics (37%) met the threshold for consensus. Domains with the greatest level of consensus were empiric therapy (11/13 topics, 85%) and duration of therapy (5/8, 63%), whereas the domains with the fewest topics achieving consensus were diagnostics (2/14, 14%) and AMR (1/18, 6%).

**Figure:**
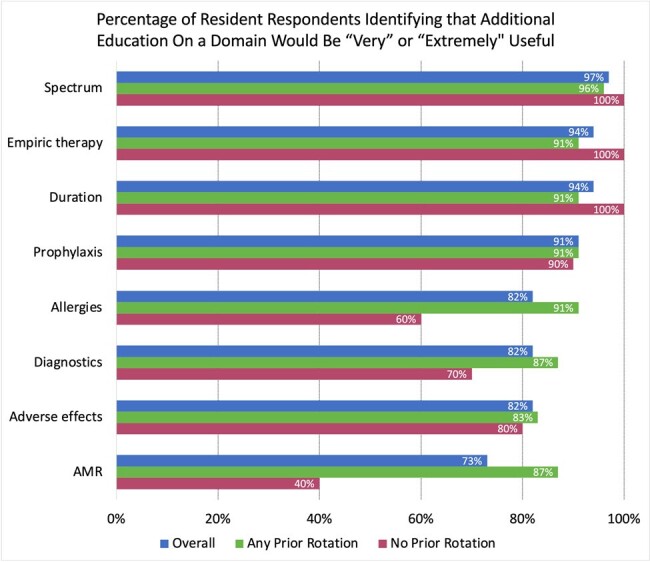

"Any prior rotation" refers to a respondent who had completed an ID or AS rotation in either medical school or residency. "No prior rotation" refers to a respondent who never completed either an ID or AS rotation at any point in training. Respondents who had previously completed an ID/AS rotation were more likely to rate additional education on AMR as “very” or extremely” useful than those who reported no prior experiences (87% versus 40%, OR 8.90, 95% CI 1.61-63.0, p=0.01). There were no other statistically significant differences in responses when comparing prior experiences or year in training for other domains.

ID: infectious diseases, AS: antimicrobial stewardship, AMR: antimicrobial resistance

**Conclusion:**

An AS curriculum for pediatric residents focused on empiric therapy and duration of therapy is most likely to meet the needs of both pediatric residents and content experts. Incorporating content expert opinions may lead to a more focused curriculum than surveying residents alone. Formal ID or AS experiences during training may improve pediatric resident perception of the usefulness of additional education on AMR.

Topics Achieving Consensus on Content Expert Survey

**Figure:**
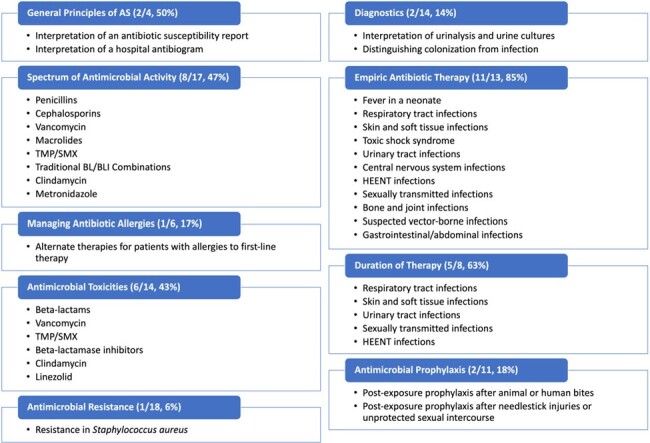

Topics are categorized by domain. Consensus was defined as ≥80% of respondents identifying a topic as "very" or "extremely" important for a graduate of a pediatric residency program to be knowledgeable on. Each header identifies the number of topics within each domain that achieved consensus, as well as the number of possible topics within each domain and the percentage of topics that achieved consensus.

**Disclosures:**

**Jason G. Newland, MD, MEd**, Moderna: Grant/Research Support|Pfizer: Grant/Research Support

